# Supplementary material for: Individual-specific functional connectivity improves prediction of Alzheimer’s disease’s symptoms in elderly people regardless of APOE ε4 genotype
Source: Commun Biol. 2023 May 31;6:581. doi: 10.1038/s42003-023-04952-6 (PMC10232409; doi:10.1038/s42003-023-04952-6)
Supplement: Supplementary file 2 — Supplementary Information [file 42003_2023_4952_MOESM2_ESM.pdf]

SUPPLEMENTARY MATERIAL

Individual-specific functional connectivity improves prediction of Alzheimer's disease's symptoms in elderly people regardless of *APOE ε4* genotype

Lin Hua, Fei Gao, Xiaoluan Xia, Qiwei Guo, Yonghua Zhao, Shaohui Huang and Zhen Yuan

Contents

Figure S1 2

Figure S2 3

Figure S3 4

Figure S4 5

URLs 6

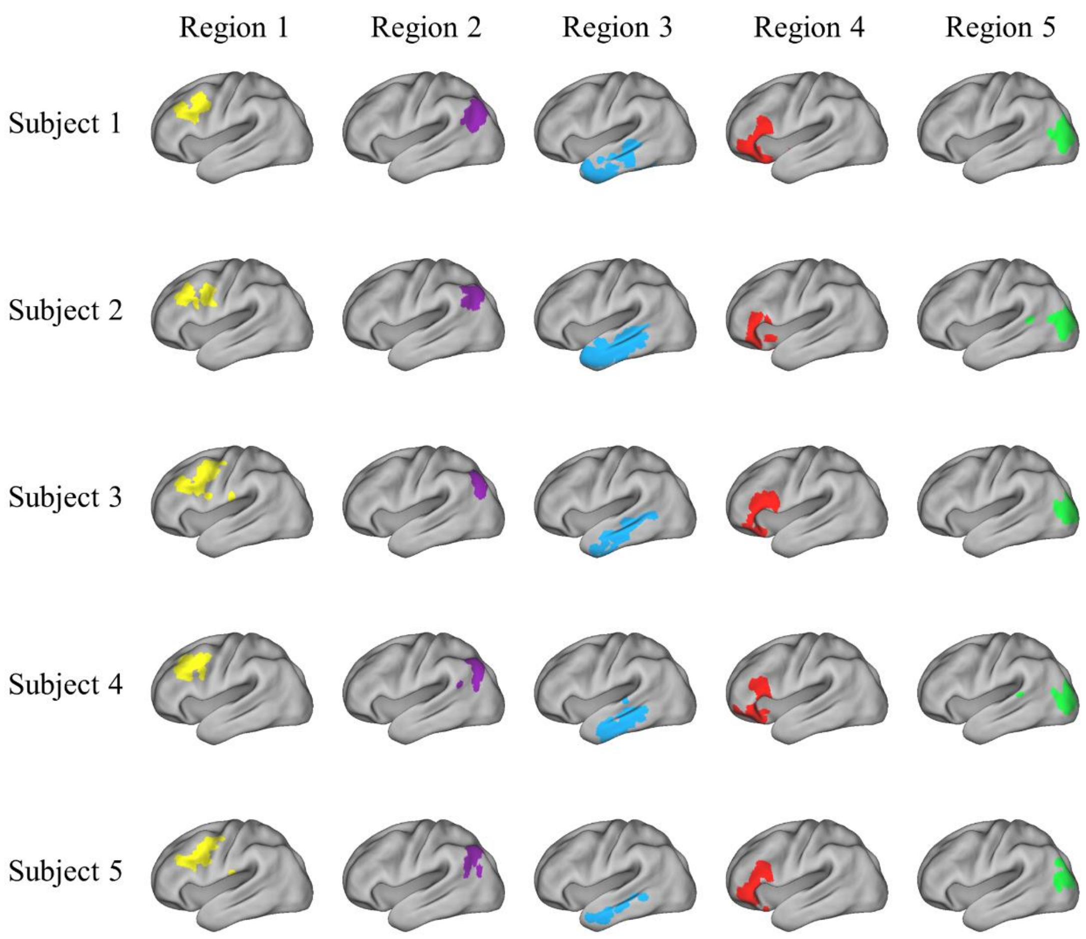

Figure S1. Five exemplary ROIs in five randomly selected participants were plotted on the brain surface. The ROIs showed marked variability in size and position. Colored regions denoted different ROIs across participants.

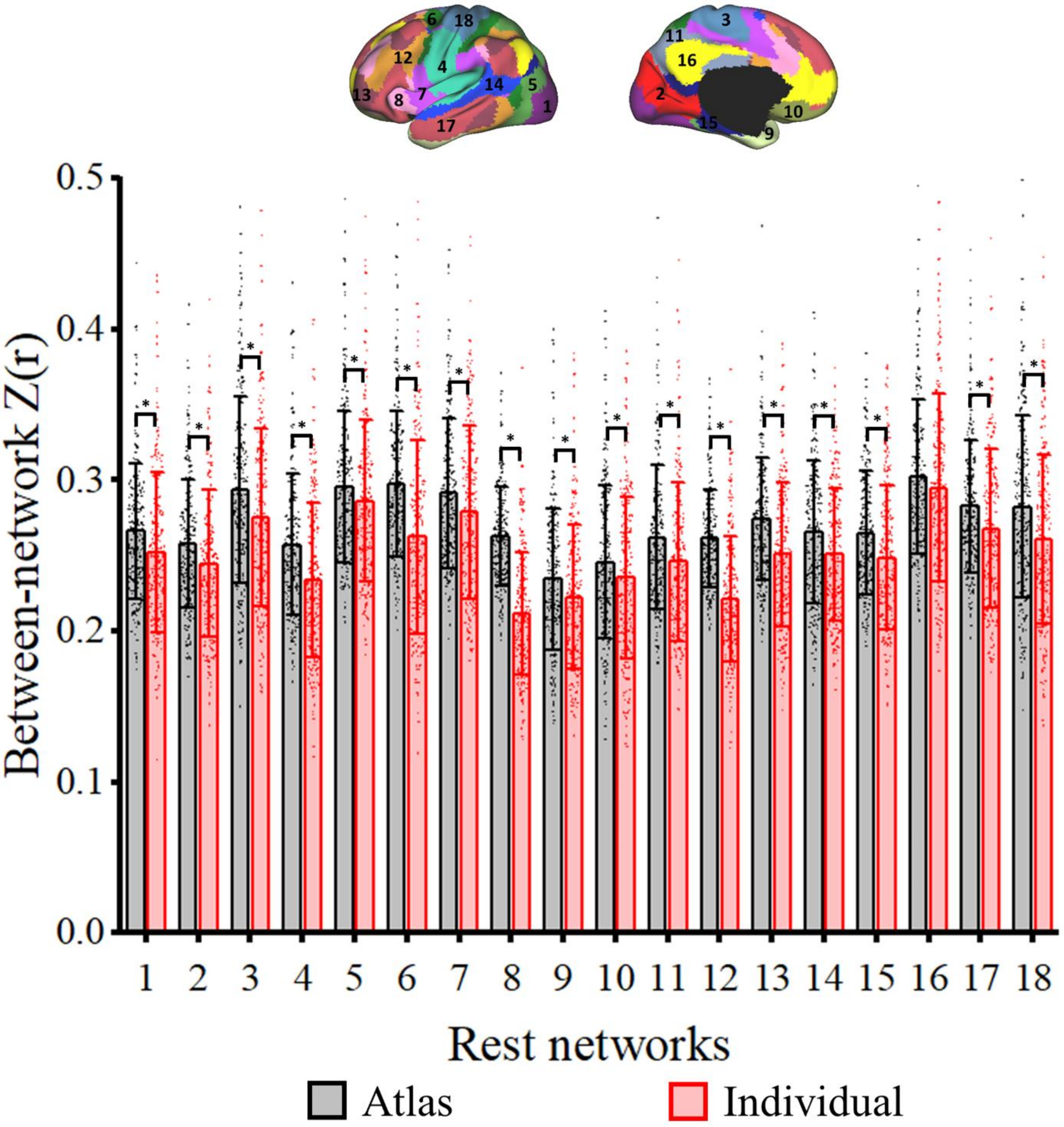

Figure S2. Functional connectivity strength between different networks was significantly reduced when the regions were individual-specific compared to atlas-based ($p < 0.01$ for 17 of the 18 networks across all participants ($N = 235$). Error bars denote standard deviation. * means $p < 0.01$, paired t-test, Bonferroni correction for 18 comparisons). Mean between-network connectivity decreased by 7.25% when the regions were individual-specific.

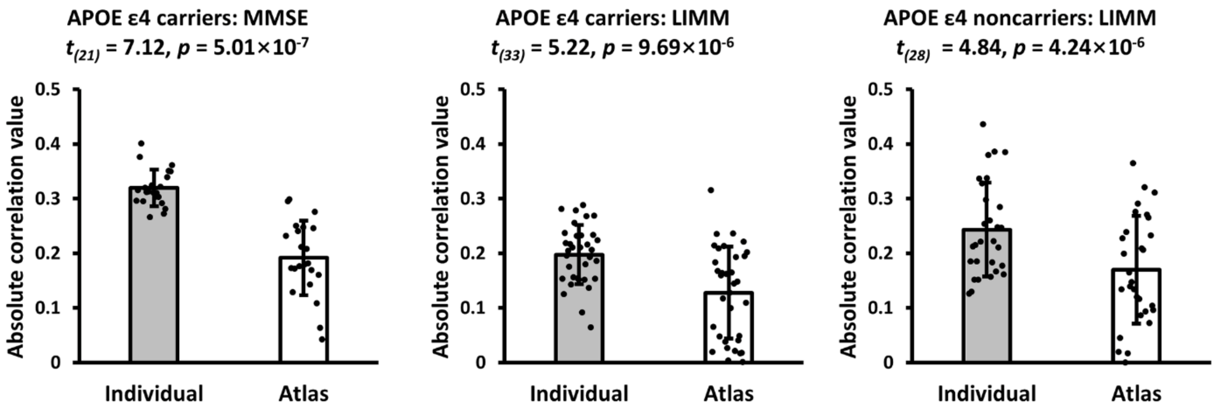

Figure S3. Individual-specific functional connections that showed a higher correlation with symptoms were redefined using the ROIs in the atlas. The same connections (N = 22, N = 34 and N = 29) showed weaker correlations (paired t-test) with all symptom scores when the connections were defined using the atlas (black) compared to connections defined in individuals (red). All p values were corrected using Bonferroni method. Error bars denote standard deviation.

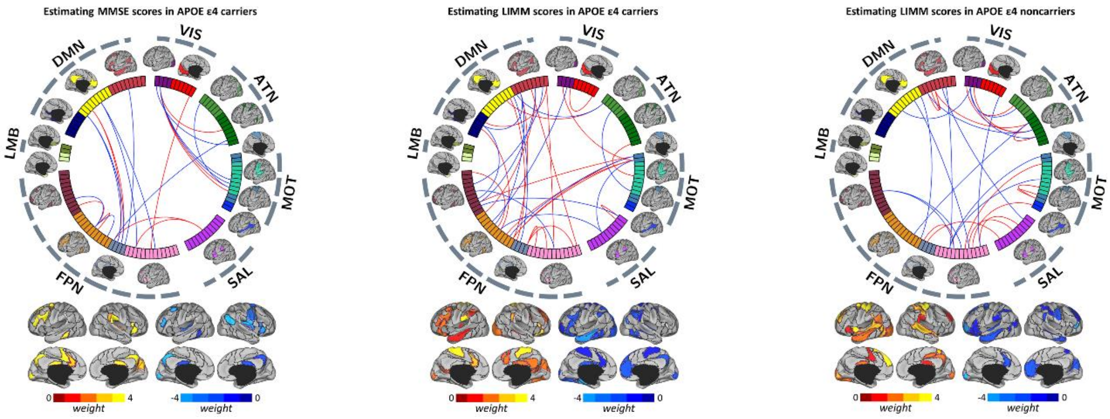

Figure S4. (A) Connections that contributed to the estimation of the MMSE scores in APOE ε4 carriers (N = 120). (B) Connections that contributed to the estimation of the LIMM scores in APOE ε4 carriers (N = 120). (C) Connections that contributed to the estimation of the LIMM scores in APOE ε4 noncarriers (N = 115). Connections that were positively correlated with the symptom scores are shown in red and connections that were negatively correlated with the symptom scores are shown in blue.

URLs

Alzheimer’s Disease Neuroimaging Initiative (ADNI; https://adni.loni.usc.edu/)

FSL (http://www.fmrib.ox.ac.uk/fsl/)

FreeSurfer (http://surfer.nmr.mgh.harvard.edu)

LIBSVM toolbox (https://www.csie.ntu.edu.tw/~cjlin/libsvm/)

LIBLINEAR toolbox (https://www.csie.ntu.edu.tw-/~cjlin/liblinear/)

CARET software (http://brainvis.wustl.edu/wiki/index.php/Caret:Download)

Circos (http://circos.ca/)
