# Supplementary material for: Individual-specific functional connectivity improves prediction of Alzheimer’s disease’s symptoms in elderly people regardless of APOE ε4 genotype
Source: Commun Biol. 2023 May 31;6:581. doi: 10.1038/s42003-023-04952-6 (PMC10232409; doi:10.1038/s42003-023-04952-6)
Supplement: Supplementary file 3 — Description of Additional Supplementary Files [file 42003_2023_4952_MOESM3_ESM.pdf]

Description of Additional Supplementary Files

File name: Supplementary Data 1

Description: The source data of Figure 2

File name: Supplementary Data 2

Description: The source data of Figure 3

File name: Supplementary Data 3

Description: The source data of Figure 4-6

File name: Supplementary Data 4

Description: The source data of Figure 7

File name: Supplementary Data 5

Description: The source data of Figure 8
