# Supplementary material for: Individual-specific functional connectivity improves prediction of Alzheimer’s disease’s symptoms in elderly people regardless of APOE ε4 genotype
Source: Commun Biol. 2023 May 31;6:581. doi: 10.1038/s42003-023-04952-6 (PMC10232409; doi:10.1038/s42003-023-04952-6)
Supplement: Supplementary file 5 — Reporting Summary [file 42003_2023_4952_MOESM5_ESM.pdf]

nature portfolio

Corresponding author(s): Zhen Yuan

Last updated by author(s): May 1, 2023

Reporting Summary

Nature Portfolio wishes to improve the reproducibility of the work that we publish. This form provides structure for consistency and transparency in reporting. For further information on Nature Portfolio policies, see our Editorial Policies and the Editorial Policy Checklist.

Statistics

For all statistical analyses, confirm that the following items are present in the figure legend, table legend, main text, or Methods section.

n/a	Confirmed	
☐	☒	The exact sample size (*n*) for each experimental group/condition, given as a discrete number and unit of measurement
☐	☒	A statement on whether measurements were taken from distinct samples or whether the same sample was measured repeatedly
☐	☒	The statistical test(s) used AND whether they are one- or two-sided<br>*Only common tests should be described solely by name; describe more complex techniques in the Methods section.*
☐	☒	A description of all covariates tested
☐	☒	A description of any assumptions or corrections, such as tests of normality and adjustment for multiple comparisons
☐	☒	A full description of the statistical parameters including central tendency (e.g. means) or other basic estimates (e.g. regression coefficient) AND variation (e.g. standard deviation) or associated estimates of uncertainty (e.g. confidence intervals)
☐	☒	For null hypothesis testing, the test statistic (e.g. *F*, *t*, *r*) with confidence intervals, effect sizes, degrees of freedom and *P* value noted<br>*Give P values as exact values whenever suitable.*
☒	☐	For Bayesian analysis, information on the choice of priors and Markov chain Monte Carlo settings
☒	☐	For hierarchical and complex designs, identification of the appropriate level for tests and full reporting of outcomes
☐	☒	Estimates of effect sizes (e.g. Cohen's *d*, Pearson's *r*), indicating how they were calculated

Our web collection on statistics for biologists contains articles on many of the points above.

Software and code

Policy information about availability of computer code

Data collection	Participants were retrieved from the phase 2 and phase 3 datasets from the Alzheimer's Disease Neuroimaging Initiative (ADNI; https://adni.loni.usc.edu/) in light of the availability of T1-weighted and resting-state functional MRI, APOE genotypes, and symptom severity assessment including MMSE and LIMM. All steps of data processing are described in the manuscript.
Data analysis	Resting-state fMRI data were processed using the FSL package. Structural MRI data were processed using the FreeSurfer package. Prediction models were constructed using the LIBSVM toolbox and the LIBLINEAR toolbox. All analysis are described in full in the manuscript.

For manuscripts utilizing custom algorithms or software that are central to the research but not yet described in published literature, software must be made available to editors and reviewers. We strongly encourage code deposition in a community repository (e.g. GitHub). See the Nature Portfolio guidelines for submitting code & software for further information.

Data

Policy information about availability of data

All manuscripts must include a data availability statement. This statement should provide the following information, where applicable:

- Accession codes, unique identifiers, or web links for publicly available datasets
- A description of any restrictions on data availability
- For clinical datasets or third party data, please ensure that the statement adheres to our policy

The data that support the findings of this study are publicly available from the ADNI dataset (https://adni.loni.usc.edu/) upon registration and compliance with the

ADNI data use policy (https://ida.loni.usc.edu/collaboration/access/appLicense.jsp).
All methods used open-source software, and all links to the relevant software are included in Methods (URLs). Code used in the analyses described in this paper is available in https://github.com/LinHuaUM/IndivCode.git

Human research participants

Policy information about studies involving human research participants and Sex and Gender in Research.

Reporting on sex and gender	There was no sex or gender based analysis in our manuscript. Sex is reported in the demographic table. Gender is not.
Population characteristics	A total of 235 elderly participants destinated into APOE ε4 carriers (N = 120) and noncarriers (N = 115) were retrieved from the phase 2 and phase 3 datasets from the ADNI in light of the availability of T1-weighted and resting-state functional MRI, APOE genotypes, and symptom severity assessment including MMSE and LIMM. According to APOE genotypes, participants were classified into two groups: 1) APOE ε4 carriers' group with at least one APOE ε4 allele (genotype ε3/ε4 and ε4/ε4), 2) APOE ε4 noncarriers' group with genotype ε3/ε3. On average, the mean age proportion was not significant ($p > 0.05$) among the NA (69.80±5.48 for APOE ε4 carriers and 70.61±5.79 for APOE ε4 noncarriers), MCI (71.87±6.18 for APOE ε4 carriers and 73.02±8.58 for APOE ε4 noncarriers), and AD (73.18±7.84 for APOE ε4 carriers and 72.00±8.02 for APOE ε4 noncarriers) groups. Meanwhile, the gender proportion was also not significantly different ($p > 0.05$) among the NA (40.48% for APOE ε4 carriers and 44.18% for APOE ε4 noncarriers), MCI (58.97% for APOE ε4 carriers and 56.41% for APOE ε4 noncarriers), and AD groups (43.59% for APOE ε4 carriers and 42.42% for APOE ε4 noncarriers).
Recruitment	ADNI patients are recruited within the north american ADNI study from specialized centers that participate in ADNI. Details on ADNI recruitment, inclusion criteria and study design can be found on the ADNI website (https://adni.loni.usc.edu/methods/documents/).
Ethics oversight	The study was approved by the institutional review boards of all participating institutions, and written informed consent was obtained from all participants or their authorized representatives. A complete listing of ADNI investigators can be found at: http://adni.loni.usc.edu/wp-content/uploads/how_to_apply/ADNI_Acknow-ledgement_List.pdf

Note that full information on the approval of the study protocol must also be provided in the manuscript.

Field-specific reporting

Please select the one below that is the best fit for your research. If you are not sure, read the appropriate sections before making your selection.

☒ Life sciences ☐ Behavioural & social sciences ☐ Ecological, evolutionary & environmental sciences

For a reference copy of the document with all sections, see nature.com/documents/nr-reporting-summary-flat.pdf

Life sciences study design

All studies must disclose on these points even when the disclosure is negative.

Sample size	A total of 235 elderly participants (120 APOE ε4 carriers and 115 APOE ε4 noncarriers) were enrolled for this study.
Data exclusions	Individuals with ε2 allele (i.e., ε2/ε2, ε2/ε4, and ε2/ε3) were excluded due to the possible protective effect. In addition, neuroimaging data of participants with excessive head motions, severe artifacts, partial brain coverage, histories of obvious head trauma, and alcohol/drug abuse were also excluded.
Replication	Findings were validated across two clinical measurements, and comparable between individual-specific FC and atlas-based FC.
Randomization	This was an observational study without any intervention, so no randomization was performed
Blinding	This was a study that retrospectively analyzed available observational clinical and neuroimaging data without any intervention, so no blinding was performed.

Reporting for specific materials, systems and methods

We require information from authors about some types of materials, experimental systems and methods used in many studies. Here, indicate whether each material, system or method listed is relevant to your study. If you are not sure if a list item applies to your research, read the appropriate section before selecting a response.

Materials & experimental systems

n/a	Involved in the study
☒	☐ Antibodies
☒	☐ Eukaryotic cell lines
☒	☐ Palaeontology and archaeology
☒	☐ Animals and other organisms
☐	☒ Clinical data
☒	☐ Dual use research of concern

Methods

n/a	Involved in the study
☒	☐ ChIP-seq
☒	☐ Flow cytometry
☐	☒ MRI-based neuroimaging

Clinical data

Policy information about clinical studies

All manuscripts should comply with the ICMJE guidelines for publication of clinical research and a completed CONSORT checklist must be included with all submissions.

Clinical trial registration: ADNI2, ClinicalTrials.gov Identifier: NCT01231971; ADNI3, ClinicalTrials.gov Identifier: NCT02854033

Study protocol: The ADNI study protocol can be found on line at https://adni.loni.usc.edu/methods/documents/

Data collection: All imaging and clinical data were collected at participating ADNI sites (between 2010-2022).

Outcomes: This study showed that compared with conventional atlas-based functional connectivity, individual-specific functional connectivity exhibited higher classification and prediction performance from normal aging to Alzheimer's disease transition in both APOE ε4 groups, while no significant performance was detected when the data of two genotyping groups were combined. Furthermore, individual-specific between-network connectivity constituted a major contributor to access cognitive symptoms.

Magnetic resonance imaging

Experimental design

Design type: Structural MRI, Resting state fMRI

Design specifications: No specific task was used

Behavioral performance measures: No behavioral measures were collected during MRI scaning

Acquisition

Imaging type(s): T1-weighted structural MRI, Resting state fMRI (Echo-Planar Imaging)

Field strength: 3T

Sequence & imaging parameters: Functional MRI (fMRI) data were selected based on the following parameters: TR = 3000 ms; TE = 30 ms; flip angle = 90o; number of slices = 48; slice thickness = 3.4 mm. The first section (200-time points, 10 minutes) of fMRI data was extracted as each participant's resting-state data.

Area of acquisition: Whole brain scan

Diffusion MRI: ☐ Used ☒ Not used

Preprocessing

Preprocessing software: Structural MRI data were processed using the FreeSurfer 7.1.1 package.
Resting-state fMRI (rs-fMRI) data were processed using the FSL 6.0 package.

Normalization: FSL

Normalization template: MNI

Noise and artifact removal: fMRI: Rigid-body correction for head motion using FSL. Framewise displacement (FD) and root-mean-square of voxel-wise differentiated signal (DVARS) were then estimated using fsl_motion_outliers implemented in FSL. Volumes with FD > 0.2 mm or DVARS > 50 were marked as outliers (censored frames). One frame before and two frames after these outliner volumes were also flagged as censored frames, together with those lasting fewer than five contiguous volumes. Volumes with more than half labeled censored frames were removed. Then, to denoise EPI images, linear regression of multiple nuisance regressors consisting of a vector of ones and linear trend, six motion correction parameters, averaged white matter signal, averaged ventricular signal, and temporal derivatives of the six motion correction parameters, averaged white matter signal, and averaged ventricular signal were regressed in the current study.

Volume censoring

To minimize the impact of motion which may compromise functional connectivity assessment, we conducted motion scrubbing. Volumes with FD > 0.2 mm or DVARS > 50 were marked as outliers (censored frames). One frame before and two frames after these outliner volumes were also flagged as censored frames, together with those lasting fewer than five contiguous volumes. Volumes with more than half labeled censored frames were removed.

Statistical modeling & inference

Model type and settings

For clinical subgroup classifications in SVM model, a significance criterion (p = 0.001) was used for feature selection between each pair of two groups. To predict each participant's symptom severity ratings in SVR model, significant features associated with symptom ratings achieving the significant criteria (p = 0.001) after Bonferroni correction were chosen to train and test the SVR model.

Effect(s) tested

We tested prediction performance between individual-specific FC and atlas-based FC among different clinical groups in elderly people with/without APOE ε4 allele, and different APOE genotyping groups in each clinical group.

Specify type of analysis: ☐ Whole brain ☒ ROI-based ☐ Both

Anatomical location(s): Individual-specific 116 ROIs and Yeo's group level atlas

Statistic type for inference (See Eklund et al. 2016)

Multivariate model

Correction

The significance of the correlation was assessed using permutation testing (1,000 permutations), which randomly reshuffled the observed symptom among the participants. The p-value was estimated by calculating the percentage of the correlation value of permutation data higher than the correlation value of real data. The p-value was corrected for multiple comparisons by using the Bonferroni method.

Models & analysis

n/a	Involved in the study
☐	☒ Functional and/or effective connectivity
☒	☐ Graph analysis
☐	☒ Multivariate modeling or predictive analysis

Functional and/or effective connectivity

Individual-specific/atlas-based functional connectivity between ROIs was constructed using the Pearson correlation coefficient between mean regional BOLD time series.

Multivariate modeling and predictive analysis

Nonlinear support vector machine (SVM) with a radial basis function (RBF) kernel was trained to assess the prediction performance of clinical subgroups among NA, MCI and AD in different genotyping groups, and genotyping groups between APOE ε4 carriers and noncarriers in different clinical subgroups.
Support vector machine for regression algorithm (L2-regularized L2-loss SVR model) was trained to predict each participant's symptom severity ratings.
